# Patrolling monocytes sensing herpes simplex virus in severe drug eruption

**DOI:** 10.1186/2045-7022-4-S3-P46

**Published:** 2014-07-18

**Authors:** Yukiko Ushigome, Ryo Takahashi, Testuo Shiohara

**Affiliations:** 1 Kyorin University School of Medicine, Department of Dermatology, Japan

## 

Drug-induced hypersensitivity syndrome/drug rash with eosinophilia and systemic symptoms (DiHS/DRESS) and Stevens-Johnson syndrome (SJS)/toxic epidermal necrolysis (TEN) are severe drug eruptions characterized by contrasting clinical pictures. Sequential reactivations of several herpesviruses have exclusively been demonstrated in the former. It remains to be determined, however, why the sequential reactivations uniquely occur in patients with DiHS/DRESS. In this regard, some clues may come from previous studies on monocytes. According to these studies, human blood monocytes can be separated into three distinct subsets: CD14+CD16- classical monocytes (cMOs), CD14+CD16+ intermediate monocytes and CD14dim CD16+ patrolling (proinflammatory) monocytes (pMOs). pMOs have been shown to patrol blood vessels and selectively detect virally infected cells to produce proinflammatory cytokines mediating anti-viral roles. In this study, we investigated the dynamics of this population in relation to other immune cells in DiHS/DRESS and SJS/TEN. Surprisingly, pMOs have been depleted from the circulation and skin lesions in the acute stage of DiHS/DRESS, while the corresponding skin lesions of SJS/TEN were characterized by massive infiltrations of pMOs: this preferential depletion of pMOs was associated with expansions of CD4+CD25+ regulatory T cells (Tregs) in DiHS/DRESS. More importantly, paired immunoglobulin-like type 2 receptor a (PILR-a) and herpesvirus entry mediator (HVEM), which can specifically bind to herpes simplex virus (HSV) envelope glycoprotein B (gB) and gD, respectively, were preferentially expressed on pMOs. pMOs infiltrated into the damaged epidermis in SJS/TEN lesions expressed HSV antigen, suggesting the role of HSV-bearing pMOs in the pathogenesis of SJS/TEN. Upon clinical resolution in DiHS/DRESS, pMOs returned to normal frequencies and Tregs thus expanded were contracted. However, the kinetics of restoration of pMOs was so distinct depending on the treatment during the acute stage: pMOs, when not treated with corticosteroids in the acute stage of DiHS/DRESS, returned to normal with a much faster kinetics than those treated with corticosteroids. Of note, rapid restoration of pMOs in patients not treated with corticosteroids led to the frequent occurrence of autoimmune sequelae. Such dynamic alterations of both populations would be responsible for the subsequent occurrence of herpesvirus reactivations that can lead to autoimmune sequelae.

